# Supplementary material for: Biological methane production under putative Enceladus-like conditions
Source: Nat Commun. 2018 Feb 27;9:748. doi: 10.1038/s41467-018-02876-y (PMC5829080; doi:10.1038/s41467-018-02876-y)
Supplement: Supplementary file 2 — Supplementary Information [file 41467_2018_2876_MOESM2_ESM.docx]

**
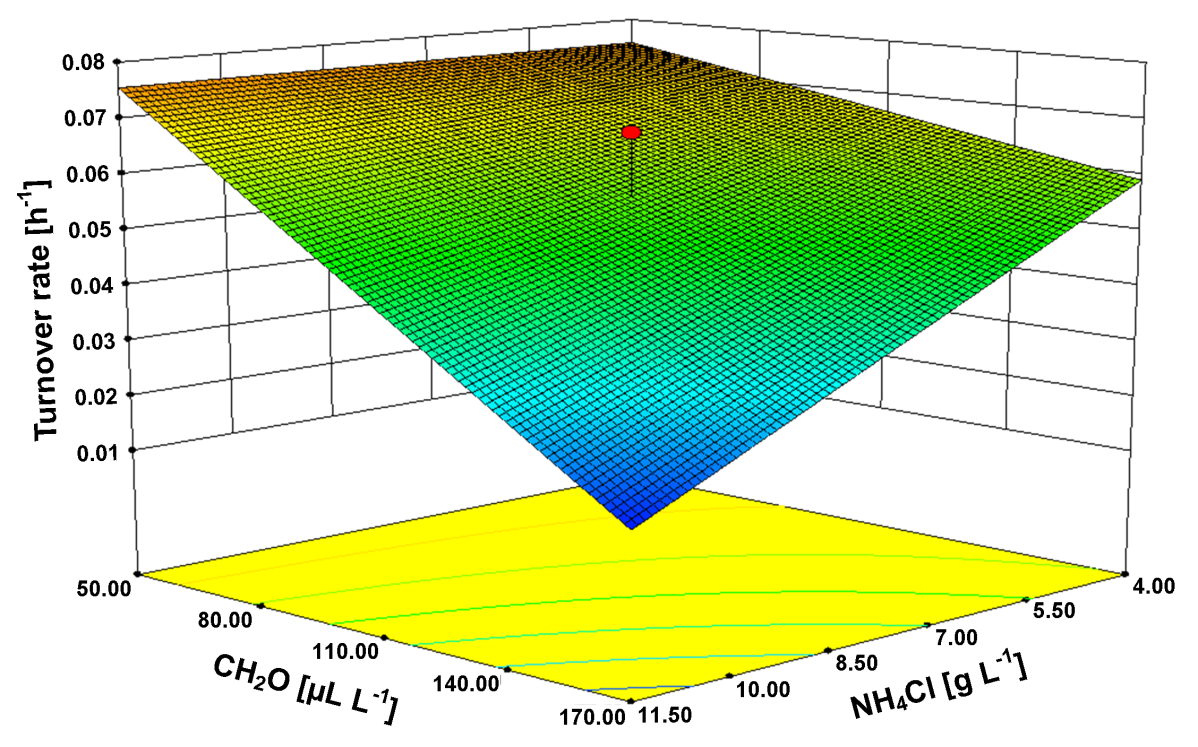
**

**Supplementary Figure 1 | Turnover rate in [h^-1^] as function of CH_2_O and NH_4_Cl concentrations.** The turnover rate reached its maximum value at low CH_2_O concentration. At high CH_2_O concentration the turnover rate is higher for low NH_4_Cl concentrations. This study was based on a DoE (see Methods and Fig. 2 and Supplementary Fig. 2 and Supplementary Tables 1 and 2). Supplementary Table 1 may be used for unit comparison.

**
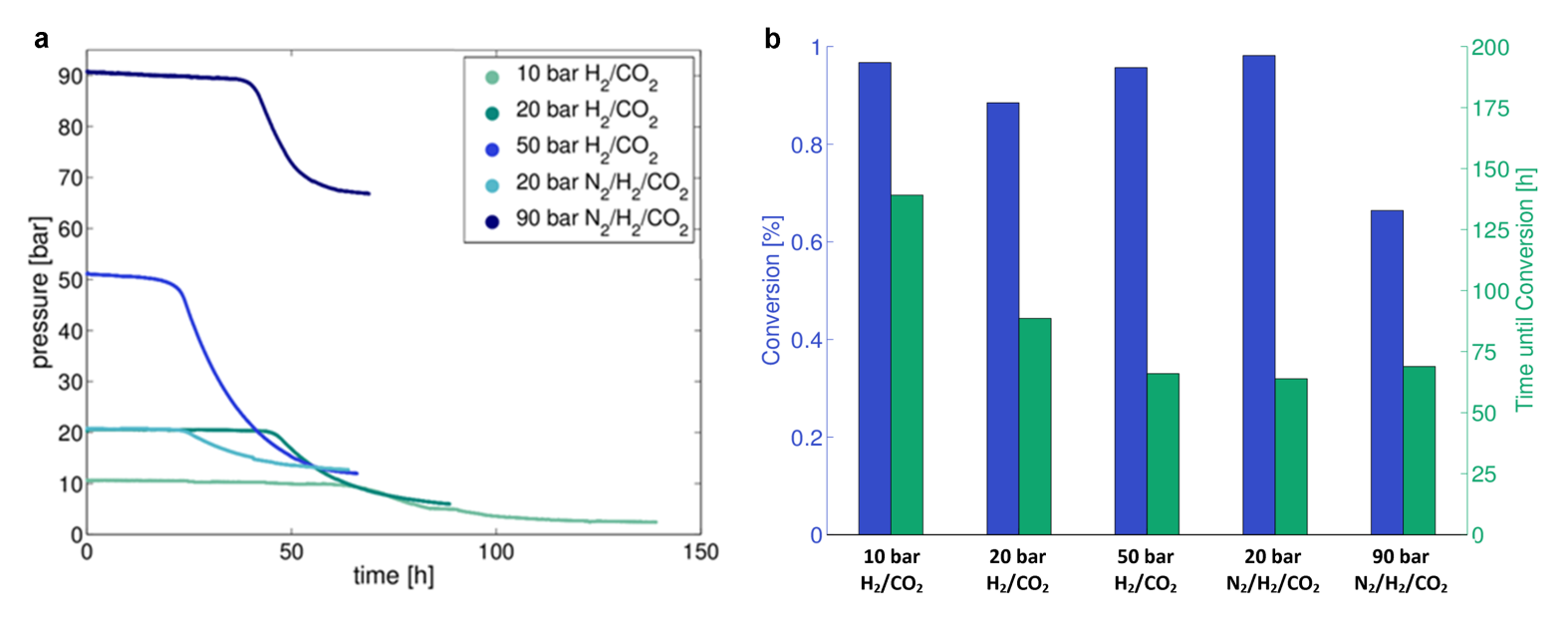
**

**Supplementary Figure 2 | H_2_/CO_2_ conversion of *M. okinawensis* under high pressure conditions.** **a,** decreasing pressure over time as indirect evidence for stoichiometric H_2_/CO_2_ to CH_4_ conversion. If 50 Vol.-% N_2_ is added to the gas phase, the time until conversion from CO_2_ to CH_4_ is even shorter (as seen for the experiments at 20 bar). **b,** the conversion (or turnover) was examined using the observed pressure drop according to equation (2) without the division by Δt.


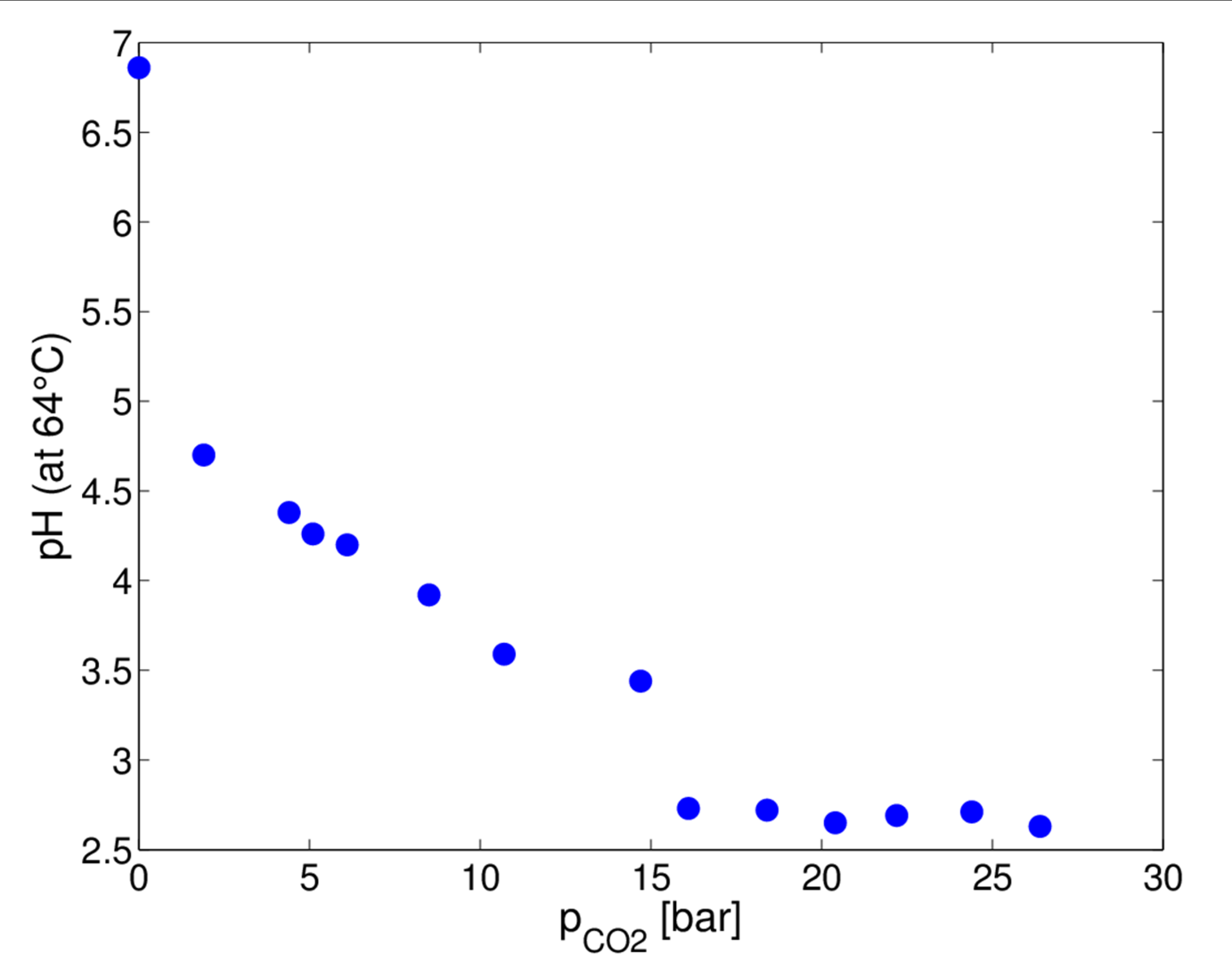


**Supplementary Figure 3 | Analysis of p_CO2_ during high pressure experiments.** The measurements were performed at 65 °C using the medium composition described in Supplementary Table 3. Already at a low p_CO2_ (1.9 bar), a pH < 5 is found.

**Supplementary Table 1 | Concentrations of liquid inhibitors in the DoE study.**

|  | **NH_4_Cl**  **[g L^-1^]** | **NH_4_Cl**  **[mol L^-1^]** | **CH_2_O (37%)**  **[µL L^-1^]** | **CH_2_O (37%)**  **[mmol L^-1^]** | **CH_3_OH**  **[µL L^-1^]** | **CH_3_OH**  **[mmol L^-1^]** |
| --- | --- | --- | --- | --- | --- | --- |
| **A** | 4.00 | 0.075 | 50.00 | 1.815 | 50.00 | 1.234 |
| **B** | 11.50 | 0.215 | 50.00 | 1.815 | 50.00 | 1.234 |
| **C** | 4.00 | 0.075 | 170.00 | 6.171 | 50.00 | 1.234 |
| **D** | 11.50 | 0.215 | 170.00 | 6.171 | 50.00 | 1.234 |
| **E** | 4.00 | 0.075 | 50.00 | 1.815 | 170.00 | 4.197 |
| **F** | 11.50 | 0.215 | 50.00 | 1.815 | 170.00 | 4.197 |
| **G** | 4.00 | 0.075 | 170.00 | 6.171 | 170.00 | 4.197 |
| **H** | 11.50 | 0.215 | 170.00 | 6.171 | 170.00 | 4.197 |
| **I** | 1.44 | 0.027 | 110.00 | 3.993 | 110.00 | 2.716 |
| **J** | 14.06 | 0.263 | 110.00 | 3.993 | 110.00 | 2.716 |
| **K** | 7.75 | 0.145 | 9.09 | 0.330 | 110.00 | 2.716 |
| **L** | 7.75 | 0.145 | 210.91 | 7.656 | 110.00 | 2.716 |
| **M** | 7.75 | 0.145 | 110.00 | 3.993 | 9.09 | 0.224 |
| **N** | 7.75 | 0.145 | 110.00 | 3.993 | 210.91 | 5.207 |
| **O** | 7.75 | 0.145 | 110.00 | 3.993 | 110.00 | 2.716 |

**Supplementary Table 2 | ANOVA results of the statistical analysis for the turnover rate in the DoE study.**

| **Source** | **Sum of Squares** | **df** | **Mean Square** | **F Value** | **p-value Prob > F** |  |
| --- | --- | --- | --- | --- | --- | --- |
|  |  |  |  |  |  |  |
| **Model** | 0.023165174 | 5 | 0.004633035 | 149.3953129 | < 0.0001 | significant |
| **A-NH_4_Cl** | 0.004846834 | 1 | 0.004846834 | 156.2894135 | < 0.0001 |  |
| **B-CH_2_O** | 0.014655254 | 1 | 0.014655254 | 472.5684829 | < 0.0001 |  |
| **C-CH_3_OH** | 2.07293∙10^-7^ | 1 | 2.07293∙10^-7^ | 0.006684316 | 0.9353 |  |
| **AB** | 0.002798073 | 1 | 0.002798073 | 90.22573942 | < 0.0001 |  |
| **C^2^** | 0.000566374 | 1 | 0.000566374 | 18.26312333 | 0.0001 |  |
| **Residual** | 0.001178453 | 38 | 3.10119∙10^-5^ |  |  |  |
| **Pure Error** | 0.00023715 | 29 | 8.1776∙10^-6^ |  |  |  |
| **Cor Total** | 0.024343627 | 43 |  |  |  |  |
|  |  |  |  |  |  |  |
| **Std. Dev.** | 0.005568834 |  | **R-Squared** | 0.951590911 |  |  |
| **Mean** | 0.059538329 |  | **Adj R-Squared** | 0.945221295 |  |  |
| **C.V. %** | 9.353359965 |  | **Pred R-Squared** | 0.935615849 |  |  |
| **PRESS** | 0.001567344 |  | **Adeq Precision** | 33.8204532 |  |  |
|  |  |  |  |  |  |  |
|  | **Coefficient** |  | **Standard** | **95% CI** | **95% CI** |  |
| **Factor** | **Estimate** | **df** | **Error** | **Low** | **High** | **VIF** |
| **Intercept** | 0.05630249 | 1 | 0.001201758 | 0.053869659 | 0.058735321 |  |
| **A-NH_4_Cl** | -0.011029395 | 1 | 0.00088224 | -0.012815397 | -0.009243393 | 1.002624948 |
| **B-CH_2_O** | -0.019178706 | 1 | 0.00088224 | -0.020964708 | -0.017392704 | 1.002624948 |
| **C-C** | -7.21299∙10^-5^ | 1 | 0.00088224 | -0.001858132 | 0.001713872 | 1.002624948 |
| **AB** | -0.011055204 | 1 | 0.001163862 | -0.01341132 | -0.008699089 | 1.00362851 |
| **C^2^** | 0.004040707 | 1 | 0.000945518 | 0.002126606 | 0.005954808 | 1.000031475 |

**Supplementary Table 3 | Liquid medium composition (incl. inhibitors) used in the low and high pressure experiments for *M. okinawensis* (for final volume of 1 L).**

| **Compound**^*^ | **Amount** |
| --- | --- |
| K_2_HPO_4_ | 0.14 g |
| CaCl_2_ x 2 H_2_O | 0.14 g |
| NH_4_Cl | 11.50 g |
| MgSO_4_ x 7 H_2_O | 3.40 g |
| MgCl_2_ x 6 H_2_O | 4.10 g |
| KCl | 0.33 g |
| NiCl_2_ x 6 H_2_O | 0.50 mg |
| Na_2_SeO_3_ x 5 H_2_O | 0.50 mg |
| NaCl | 30.00 g |
| FeSO_4_ x 7 H_2_O | 7 mg |
| NaHCO_3_ | 1.00 g |
| L-Cysteine-HCl x H_2_O | 0.50 g |
| Na_2_S x 9 H_2_O | 0.50 g |
| H_2_CO (37%) | 0.11 mL |
| CH_3_OH | 0.16 mL |
| Trace Element Solution  (DSMZ Medium 141) | 10.00 mL |

| ddH_2_O | ad 1000mL |
| --- | --- |

^*^ The medium composition is similar to DSMZ medium 282 (for *Methanococcus jannaschii*).

**Supplementary Table 4 | Results of serpentinization models at 50 bar.**

| **Model** | **pH** | **H_2_ production rate [nmol g^-1^ L^-1^ d^-1^]** | **CH_4_ production rate [nmol g^-1^ L^-1^ d^-1^]** | **water : rock ratio** | **Moles H_2_ produced per mole olivine** |
| --- | --- | --- | --- | --- | --- |
| **Model 1: Fo_90_* : En : Diop = 8 : 1 : 1** | | | | |  |
| 25 °C | 12.2 | 0.138 | 0.064 | 0.126 | 0.002 |
| 50 °C | 11.3 | 4.58 | 2.05 | 0.126 | 0.002 |
| 100 °C | 9.82 | 49.2 | 19.2 | 0.126 | 0.003 |
| **Model 2: Fo_90_*** | | | | |  |
| 25 °C | 9.33 | 0.250 | 0.056 | 0.124 | 0.004 |
| 50 °C | 8.60 | 4.03 | 1.07 | 0.124 | 0.004 |
| 100 °C | 7.45 | 80.4 | 16.9 | 0.124 | 0.004 |
| **Model 3: Fo_50_^†^** | | | | |  |
| 25 °C | 8.12 | 8.68 | 0.209 | 0.104 | 0.033 |
| 50 °C | 7.50 | 34.7 | 1.35 | 0.104 | 0.033 |
| 100 °C | 6.50 | 517 | 12.5 | 0.104 | 0.032 |
| **Model 4: Fo_20_^‡^** | | | | |  |
| 25 °C | 7.94 | 12.7 | 0.184 | 0.094 | 0.054 |
| 50 °C | 7.29 | 50.7 | 1.32 | 0.094 | 0.053 |
| 100 °C | 6.26 | 757 | 11.0 | 0.094 | 0.052 |

*Fo_90_ = Forsteritic olivine (Forsterite:Fayalaite = 9:1), En = Enstatite, Diop = Diopside

**^†^**Fo_50_ = Forsteritic olivine (Forsterite:Fayalaite = 1:1)

^‡^Fo_20_ = Fayalitic olivine (Forsterite:Fayalaite = 2:8)

**Supplementary Table 5 | Liquid medium composition (“standard medium”) used in the low pressure for *M. marburgensis* experiments (for final volume of 1 L).**

| **Compound** | **Amount** |
| --- | --- |
| KH_2_PO_4_ | 6.8 g |
| NH_4_Cl | 2.1 g |
| Na_2_CO_3_ | 3.6 g |
| Na_2_S x 9 H_2_O | 0.25 g |
| Trace Element Solution (see Suppl. Tab.7) | 10.00 mL |
| ddH_2_O | ad 1000 mL |

**Supplementary Table 6 | Liquid medium composition (“Medium w/o NaHCO_3_”) used in the low pressure experiments for *M. marburgensis* (for final volume of 1 L).**

| **Compound** | **Amount** |
| --- | --- |
| KH_2_PO_4_ | 6.8 g |
| NH_4_Cl | 2.1 g |
| NaCl | 1.0 g |
| Na_2_S x 9 H_2_O | 0.25 g |
| with 10M NaOH adjust to pH=6.8 | |
| Trace Element Solution (see Suppl. Tab.7) | 10.00 mL |
| ddH_2_O | ad 1000 mL |

**Supplementary Table 7 | Composition of the trace element solution used in the low pressure experiments for *M. marburgensis* (for final volume of 1 L).**

| **Compound** | **Amount** |
| --- | --- |
| Titriplex I | 9.0 g |
| ddH_2_O | 800 mL |
| with 5M NaOH adjust to pH=6.5, then add following ingredients: | |
| MgCl_2_ x 6H_2_O | 4.0 g |
| FeCl_2_ x 4H_2_O | 1.0 g |
| CoCl_2_ x 6H_2_O | 20 mg |
| NiCl_2_ x 6H_2_O | 120 mg |
| NaMoO_4_ x 2H_2_O | 20 mg |
| with 1M NaOH adjust to pH=7.0 | |
| ddH_2_O | ad 1000 mL |

**Supplementary Table 8 | Liquid medium composition (“standard medium”) used in the low pressure experiments for *M. villosus* (18 g NaCl) and *M. okinawensis* (30 g NaCl) (for final volume of 1 L).**

| **Compound**^*^ | **Amount** |
| --- | --- |
| K_2_HPO_4_ | 0.14 g |
| CaCl_2_ x 2 H_2_O | 0.14 g |
| NH_4_Cl | 0.25 g |
| MgSO_4_ x 7 H_2_O | 3.40 g |
| MgCl_2_ x 6 H_2_O | 4.10 g |
| KCl | 0.33 g |
| NiCl_2_ x 6 H_2_O | 0.50 mg |
| Na_2_SeO_3_ x 5 H_2_O | 0.50 mg |
| NaCl | 18.00 g / 30.00 g |
| FeSO_4_ x 7 H_2_O | 7 mg |
| NaHCO_3_ | 1.00 g |
| L-Cysteine-HCl x H_2_O | 0.50 g |
| Na_2_S x 9 H_2_O | 0.50 g |
| Vitamin Solution (DSMZ Medium 141) | 10.00 mL |
| Trace Element Solution  (DSMZ Medium 141) | 10.00 mL |
| ddH_2_O | ad 1000 mL |

^*^ The medium composition is similar to DSMZ medium 282 (for *Methanococcus jannaschii*).

**Supplementary Table 9 | Liquid medium composition (“Medium w/o NaHCO_3_”) used in the low pressure experiments (for final volume of 1 L) for *M. villosus* (18.7 g NaCl) and *M. okinawensis* (30.7 g NaCl).**

| **Compound**^*^ | **Amount** |
| --- | --- |
| K_2_HPO_4_ | 0.14 g |
| CaCl_2_ x 2 H_2_O | 0.14 g |
| NH_4_Cl | 0.25 g |
| MgSO_4_ x 7 H_2_O | 3.40 g |
| MgCl_2_ x 6 H_2_O | 4.10 g |
| KCl | 0.33 g |
| NiCl_2_ x 6 H_2_O | 0.50 mg |
| Na_2_SeO_3_ x 5 H_2_O | 0.50 mg |
| NaCl | 18.70 g / 30.70 g |
| FeSO_4_ x 7 H_2_O | 7 mg |
| L-Cysteine-HCl x H_2_O | 0.50 g |
| Na_2_S x 9 H_2_O | 0.50 g |
| Vitamin Solution (DSMZ Medium 141) | 10.00 mL |
| Trace Element Solution  (DSMZ Medium 141) | 10.00 mL |
| ddH_2_O | ad 1000 mL |

^*^ The medium composition is similar to DSMZ medium 282 (for *Methanococcus jannaschii*).

**Supplementary Table 10 | Liquid medium composition (“Medium w/o Vitamins”) used in the low pressure experiments for *M. villosus* (18 g NaCl) and *M. okinawensis* (30 g NaCl) and in the initial high pressure experiments for *M. okinawensis* (30 g NaCl) (for final volume of 1 L).**

| **Compound**^*^ | **Amount** |
| --- | --- |
| K_2_HPO_4_ | 0.14 g |
| CaCl_2_ x 2 H_2_O | 0.14 g |
| NH_4_Cl | 0.25 g |
| MgSO_4_ x 7 H_2_O | 3.40 g |
| MgCl_2_ x 6 H_2_O | 4.10 g |
| KCl | 0.33 g |
| NiCl_2_ x 6 H_2_O | 0.50 mg |
| Na_2_SeO_3_ x 5 H_2_O | 0.50 mg |
| NaCl | 18.00 g / 30.00 g |
| FeSO_4_ x 7 H_2_O | 7 mg |
| NaHCO_3_ | 1.00 g |
| L-Cysteine-HCl x H_2_O | 0.50 g |
| Na_2_S x 9 H_2_O | 0.50 g |
| Trace Element Solution  (DSMZ Medium 141) | 10.00 mL |
| ddH_2_O | ad 1000 mL |

^*^ The medium composition is similar to DSMZ medium 282 (for *Methanococcus jannaschii*).

**Supplementary Methods:**

In the following section, the MATLAB^©^ code used for the estimations of Enceladus’ interior structure is given (2 methods).

function []=pressure(c,d,s) %c=core radius, d=thickness of the ocean, s=steps

d=d*1000;

c=c*1000;

r1=1:s:c;

r2=c+1:s:c+1+d;

r3=c+2+d:s:252100;

M=107944591230692000000; %total mass in kg, source: https://solarsystem.nasa.gov/planets/enceladus/facts

G=6.67408*10^(-11); %gravitational constant

rho1=2300:1:2550; %range for core density

rho2=960:1:1080; %range for ocean density

rho3=850:1:960; %range for ice crust density

for i=1:length(rho1)

for j=1:length(r1)

M1(i,j)=4/3*pi.*r1(j)^3*rho1(i); % < core mass

g1(i,j)=G*M1(i,j)/r1(j)^2; % < core gravity acceleration

end

end

M1max=M1(:,end);

for i=1:length(rho2)

for j=1:length(r2)

for k=1:length(M1max)

M2(i,j,k)=M1max(k)+4/3*pi*(r2(j)^3-r2(1)^3)*rho2(i); % < core + ocean mass

g2(i,j,k)=G*M2(i,j,k)/r2(j)^2; % < core + ocean gravity acceleration

end

end

end

M2max=M2(:,end,:);

x=size(M2max);

x1=x(1);

x2=x(end);

for i=1:length(rho3)

for j=1:length(r3)

for k=1:x1

for l=1:x2

M3(i,j,k,l)=M2max(k,1,l)+4/3*pi*(r3(j)^3-r3(1)^3)*rho3(i); % < total mass

g3(i,j,k,l)=G*M3(i,j,k)/r3(j)^2; % < total gravity acceleration

end

end

end

end

M_total1=M*0.999999;

M_total2=M*1.000001;

maxM3=M3(:,end,:,:);

[a,b,c,d] = ind2sub(size(maxM3),find(maxM3(:,end,:,:)>M_total1 & maxM3(:,end,:,:)<M_total2)); %to find possible combination for the densities

for i=1:length(a)

Mend(i)=maxM3(a(i),b(i),c(i),d(i));

rho1end(i)=rho1(d(i));

rho2end(i)=rho2(c(i));

rho3end(i)=rho3(a(i));

for j=1:length(r1)

g1end(i,j)=g1(d(i),j);

end

for j=1:length(r2)

g2end(i,j)=g2(c(i),j,d(i));

end

for j=1:length(r3)

g3end(i,j)=g3(a(i),j,c(i),d(i));

end

end

%Method 1:

for i=1:length(a) %to determine the pressure at the water/ice boundary

for j=1:length(r3)

p3(i,j)=2/3*G*pi*rho3end(i)^2*(r3(end)^2-r3(end-j+1)^2);

end

end

for i=1:length(a) to determine the pressure at the core/water boundary

for j=1:length(r2)

p2(i,j)=p3(i,end)+2/3*G*pi*rho2end(i)^2*(r2(end)^2-r2(end-j+1)^2);

end

end

p_ocean_core1 = p2(:,end) % pressure at the core/water boundary

%Method 2:

for i=1:length(a) %to determine the pressure at the water/ice boundary

for j=1:length(r3)

p_ice(i,j)=rho3end(i)*g3end(i,j)*1000;

end

p_ice_ocean(i)=sum(p_ice(i,:));

end

for i=1:length(a) %to determine the pressure at the core/water boundary

p_water(i,1)=p_ice_ocean(i);

for j=2:length(r2)+1

p_water(i,j)=rho2end(i)*g2end(i,j-1)*1000;

end

p_ocean_core2(i)=sum(p_water(i,:));

end

p_ocean_core2 % pressure at the core/water boundary

end
